# Dehydroxyhispolon Methyl Ether, A Hispolon Derivative, Inhibits WNT/β-Catenin Signaling to Elicit Human Colorectal Carcinoma Cell Apoptosis

**DOI:** 10.3390/ijms21228839

**Published:** 2020-11-22

**Authors:** Hueng-Chuen Fan, Ya-Chu Hsieh, Li-Hsuan Li, Ching-Chin Chang, Karolína Janoušková, Modukuri V. Ramani, Gottumukkala V. Subbaraju, Kur-Ta Cheng, Chia-Che Chang

**Affiliations:** 1 Department of Pediatrics, Department of Medical Research, Tungs’ Taichung Metroharbor Hospital, Wuchi, Taichung 43503, Taiwan; fanhuengchuen@yahoo.com.tw; 2 Department of Rehabilitation, Jen-Teh Junior College of Medicine, Nursing and Management, Miaoli 35664, Taiwan; 3 Ph.D. Program in Translational Medicine, National Chung Hsing University, Taichung 40227, Taiwan; 4 Ph.D. Program in Tissue Engineering and Regenerative Medicine, National Chung Hsing University, Taichung 40227, Taiwan; asdf789515@gmail.com (Y.-C.H.); neb830414@gmail.com (L.-H.L.); 5 Institute of Biomedical Sciences, National Chung Hsing University, Taichung 40227, Taiwan; z091938882747@gmail.com (C.-C.C.); janousko@vscht.cz (K.J.); 6 University of Chemistry and Technology, 166 28 Prague, Czech Republic; 7 Department of Organic Chemistry, Andhra University, Visakhapatnam 530 003, India; ramani_v@yahoo.com (M.V.R.); subbarajugv@gmail.com (G.V.S.); 8 Department of Biochemistry and Molecular Cell Biology, Taipei Medical University, Taipei 11031, Taiwan; 9 Department of Life Sciences, The iEGG and Animal Biotechnology Research Center, Rong Hsing Research Center for Translational Medicine, National Chung Hsing University, Taichung 40227, Taiwan; 10 Traditional Herbal Medicine Research Center, Taipei Medical University Hospital, Taipei 11031, Taiwan; 11 Department of Medical Research, China Medical University Hospital, Taichung 40447, Taiwan; 12 Department of Biotechnology, Asia University, Taichung 41354, Taiwan

**Keywords:** dehydroxyhispolon methyl ether, hispolon, hispolon derivatives, *Phellinus linteus*, WNT/β-catenin, colorectal cancer

## Abstract

Colorectal cancer (CRC) is the fourth leading cause of cancer mortality worldwide. Aberrant activation of WNT/β-catenin signaling present in the vast majority of CRC cases is indispensable for CRC initiation and progression, and thus is a promising target for CRC therapeutics. Hispolon is a fungal-derived polyphenol with a pronounced anticancer effect. Several hispolon derivatives, including dehydroxyhispolon methyl ether (DHME), have been chemically synthesized for developing lead molecules with stronger anticancer activity. Herein, a DHME-elicited anti-CRC effect with the underlying mechanism is reported for the first time. Specifically, DHME was found to be more cytotoxic than hispolon against a panel of human CRC cell lines, while exerting limited toxicity to normal human colon cell line CCD 841 CoN. Additionally, the cytotoxic effect of DHME appeared to rely on inducing apoptosis. This notion was evidenced by DHME-elicited upregulation of poly (ADP-ribose) polymerase (PARP) cleavage and a cell population positively stained by annexin V, alongside the downregulation of antiapoptotic B-cell lymphoma 2 (BCL-2), whereas the blockade of apoptosis by the pan-caspase inhibitor z-VAD-fmk attenuated DHME-induced cytotoxicity. Further mechanistic inquiry revealed the inhibitory action of DHME on β-catenin-mediated, T-cell factor (TCF)-dependent transcription activity, suggesting that DHME thwarted the aberrantly active WNT/β-catenin signaling in CRC cells. Notably, ectopic expression of a dominant–active β-catenin mutant (∆N90-β-catenin) abolished DHME-induced apoptosis while also restoring BCL-2 expression. Collectively, we identified DHME as a selective proapoptotic agent against CRC cells, exerting more potent cytotoxicity than hispolon, and provoking CRC cell apoptosis via suppression of the WNT/β-catenin signaling axis.

## 1. Introduction

Colorectal cancer (CRC) is the third most frequently diagnosed and the fourth most lethal human cancer globally [1]. Aging, dietary habits of high-income countries, and lifestyle factors such as obesity, smoking, and lack of physical exercise are well-established risk factors that increase CRC incidence [2]. The high mortality rate of CRC is largely attributed to the advanced stage of disease when first diagnosed in most CRC cases, combined with the limited response to traditional chemotherapy [3]. Thus, increased nationwide screening and developing effective CRC therapeutics are indispensable measures to lower the incidence and mortality of this dreadful disease.

Accumulating evidence has delineated a number of signaling pathways whose deregulation contributes to CRC pathogenesis, including epidermal growth factor receptor (EGFR)/mitogen-activated protein kinase (MAPK), Hedgehog, Notch, phosphoinositide 3-kinase/protein Kinase B (PI3K/AKT), transforming growth factor-β (TGF-β), and WNT/β-catenin [3,4]. Notably, the canonical WNT/β-catenin signaling pathway appears to be the foremost CRC driver, as evidenced by its aberrant activation present in the vast majority of CRC cases [2,5]. In detail, the aberrant activation of WNT/β-catenin signaling in CRC cells is predominantly caused by the stabilization of β-catenin protein, as a result of inactivating mutations in the *APC* gene or activating mutations in the β-catenin-encoding gene *CTNNB1* [6]. Consequently, β-catenin is accumulated in the cytosol, allowing its nuclear translocation to upregulate T-cell factor/lymphoid enhancer factor (TCF/LEF)-dependent transcription of WNT target genes for promoting cell proliferation, survival, and invasion, along with initiating and maintaining the stemness of CRC stem cells [5,6,7,8]. Importantly, it has been confirmed that genetic or pharmacological ablation of aberrant WNT/β-catenin signaling impedes CRC cell growth in both in vitro and in vivo models, highlighting the WNT/β-catenin signaling pathway as a promising target for developing novel CRC therapeutics [6,7,8,9].

Hispolon is a polyphenolic compound structurally analogous to curcumin, and is a bioactive constituent present in the fruiting body and mycelium of medicinal mushroom *Phellinus linteus* [10,11]. Previous studies have revealed that hispolon exhibits a broad range of health beneficial effects, including antioxidant, anti-inflammatory, antiviral, antidiabetic, and anticancer [11,12,13,14]. In particular, the anticancer action of hispolon involves antiproliferation via the arrest of cell-cycle progression, induction of apoptosis, and inhibition of metastasis [15,16,17]. Given the potential of hispolon as an anticancer agent, Balaji et al. designed and chemically synthesized a panel of hispolon derivatives that were subjected to an evaluation of in vitro cytotoxicity, in the hope of finding lead molecules with more potent anticancer activity [18].

In the present study, we investigated the anti-CRC effect and the underlying mechanisms of dehydroxyhispolon methyl ether (DHME), a hispolon derivative designated as V5 in Balaji et al. [18]. Our findings provide the first evidence supporting the selective cytotoxicity of DHME, indicating stronger cytotoxicity of DHME than hispolon, revealing DHME as a proapoptotic agent, and specifying DHME as an inhibitor targeting the WNT/β-catenin–B-cell lymphoma 2 (BCL-2) pro-survival signaling axis to induce CRC cell apoptosis.

## 2. Results

### 2.1. DHME Selectively Induced CRC Cell Death while Sparing Normal Colon Epithelial Cells

**Figure 1:**
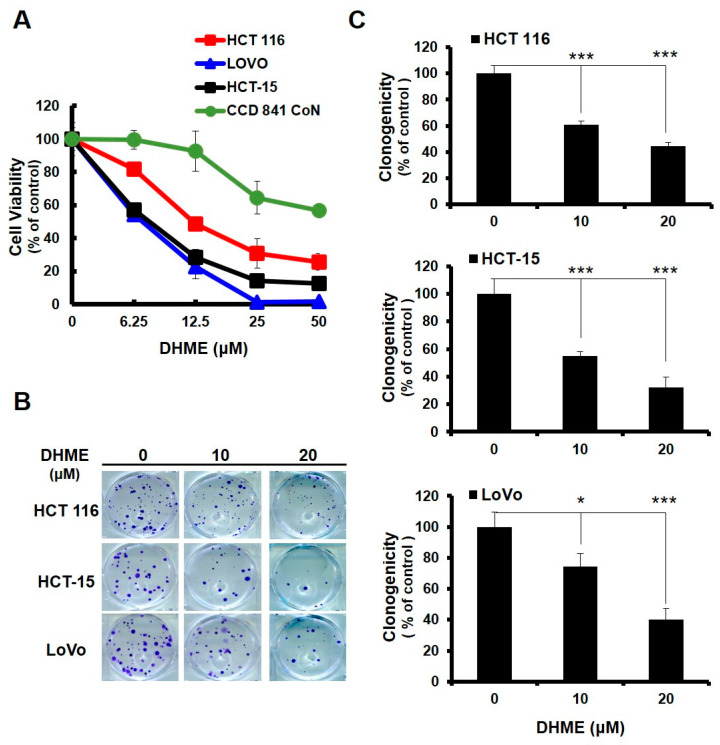
Anti-colorectal cancer (CRC) effect of dehydroxyhispolon methyl ether (DHME). (**A**) Selective cytotoxicity of DHME against malignant but not normal human colorectal epithelial cell lines. A panel of human CRC cell lines, such as HCT 116, HCT-15, and LoVo, in addition to one normal human colorectal epithelial cell line, CCD 841 CoN, were treated with DHME for 48 h, followed by cell viability evaluation using MTS assay. (**B**) DHME suppresses CRC cells to form colonies. A total of 2 × 10^2^ of human CRC cells, after 24 h of treatment with DHME, were allowed to grow in drug-free media for 10 days to form colonies, which were visualized by crystal violet staining. (**C**) Quantitative analysis of DHME-induced suppression of CRC clonogenicity. Colonies displayed in (B) were scored, and the results were subjected to statistical analysis. * *p* < 0.05; *** *p* < 0.001.

The potential cytotoxic effect of DHME on CRC cells was first examined. A panel of human colorectal carcinoma cell lines, including HCT 116, HCT-15, and LoVo, along with a normal human colon epithelial cell line CCD 841 CoN, were treated with graded doses of DHME (0~50 μM) for 48 h, followed by cell viability determination using an MTS assay. It was noticed that DHME curtailed the survival of all tested CRC cell lines in a dose-dependent way, with IC_50_ values of 12.25 ± 1.20 μM, 7.73 ± 0.25 μM, and 7.13 ± 0.35 μM for HCT 116, HCT-15, and LoVo cells, respectively. However, the viability of the CCD 841 CoN cells failed to drop to 50%, even when treated with 50 μM of DHME (Figure 1A). Apparently, DHME-induced cytotoxicity was selective to malignant rather than normal colorectal epithelial cells. To further validate DHME’s cytotoxic effect on CRC cells, the clonogenicity of DHME-treated CRC cells was evaluated. We observed a clear, dose-dependent reduction in the capacity of DHME-treated CRC cells to form colonies (Figure 1B). Specifically, compared to drug-free controls, DHME at 20 μM lowered the clonogenicity of HCT 116, HCT-15, and LoVo cells to 44.36% ± 2.69%, 32.17% ± 7.71%, and 40.09% ± 8.24% (*p* < 0.001), respectively (Figure 1C). Overall, these findings revealed a marked and selective cytotoxic effect of DHME on human CRC cells, while sparing normal colorectal epithelial cells.

### 2.2. DHME Was More Potent than Hispolon Regarding CRC Cytotoxicity

**Figure 2:**
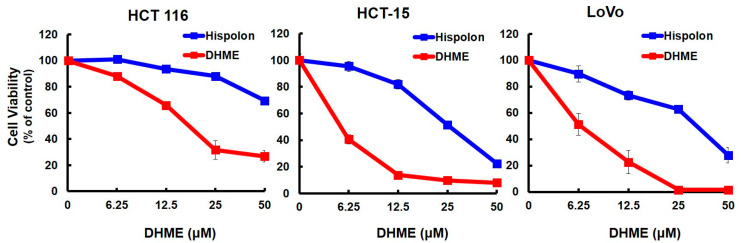
DHME is more potent than hispolon in inducing CRC cell death. HCT 116, HCT-15, and LoVo cells were treated with indicated concentration (0.00, 6.25, 12.50, and 50.00 μM) of hispolon or DHME for 48 h, and the viability of drug-treated cells was determined by MTS assay thereafter.

Hispolon was previously shown to be cytotoxic against human CRC cell lines HCT 116 and S1 [18]. Herein, the cytotoxic effects of hispolon and DHME on HCT 116, HCT-15, and LoVo cells were examined simultaneously. As shown in Figure 2, it was clearly noted that Hispolon reduced 50% of HCT-15 cell viability at 26.48 ± 1.88 μM, whereas it took only 5.59 ± 0.34 μM of DHME to achieve the same level of cytotoxicity (Figure 1A). Likewise, the IC_50_ of hispolon for LoVo cells was 34.18 ± 1.93 μM, almost a six-fold increase compared to that of DHME (6.41 ± 0.80 μM). The difference between hispolon and DHME in HCT 116 cytotoxicity was even more obvious: while hispolon at 50 μM failed to lower HCT 116 cell viability to 50%, the IC_50_ of DHME was 10.56 ± 0.75 μM (Figure 2). Clearly, our data revealed that each tested CRC cell line was much more susceptible to the cytotoxicity induced by DHME than that induced by hispolon (Figure 2).

### 2.3. DHME Was Proapoptotic to Human CRC Cells

**Figure 3:**
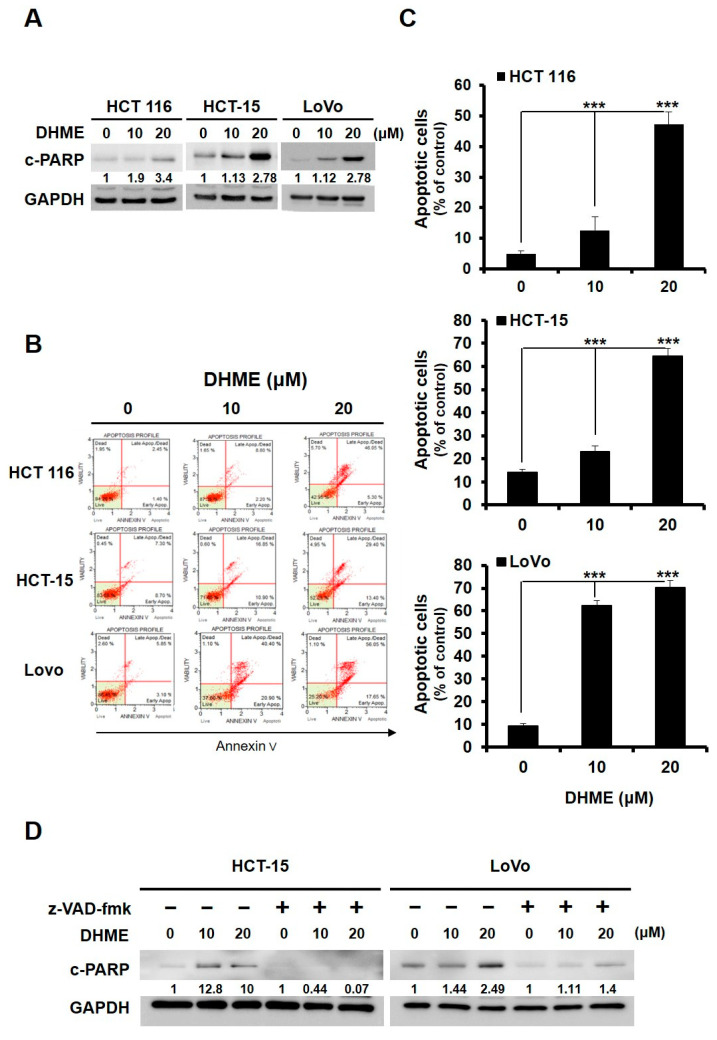

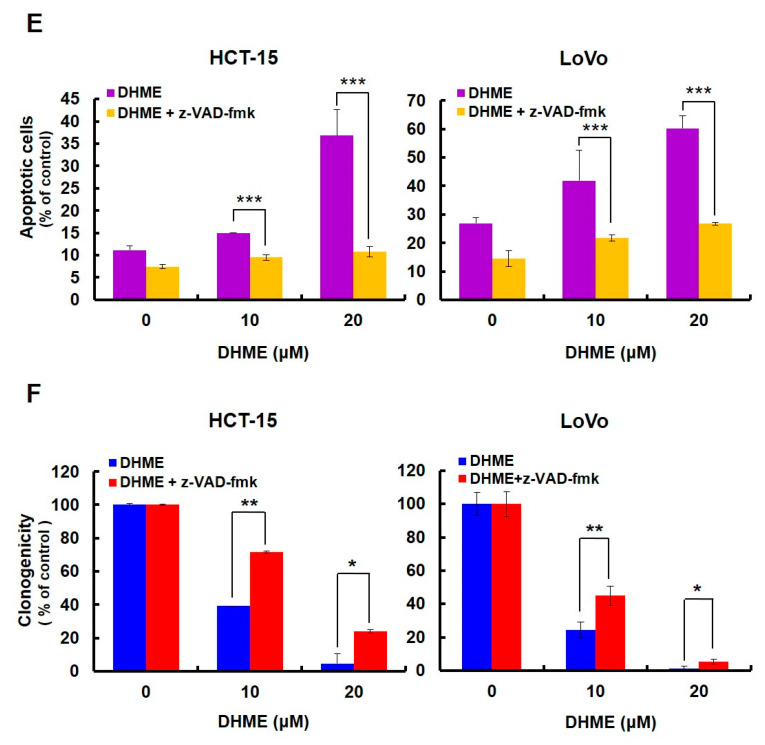
Apoptosis induction is essential for the anti-CRC action of DHME. (**A**) DHME induces poly (ADP-ribose) polymerase (PARP) cleavage. HCT 116, HCT-15, and LoVo cells were treated with DHME (0, 10, or 20 μM) for 24 h, followed by immunoblotting for the levels of cleaved PARP (c-PARP). The levels of glyceraldehyde-3-phosphate dehydrogenase (GAPDH) served as the loading control. (**B**) DHME enhances the levels of annexin V-positive cell population. Human CRC cell lines treated with DHME (0, 10, 20 μM) for 24 h were subjected to annexin V/Propidium iodide (PI) dual staining using flow cytometry analysis. Annexin V-positive cells were regarded as cells undergoing apoptosis. The levels of the cell population in each quadrant were expressed as the percentage of total cell population. The horizontal axis denotes the intensity of annexin V, and the vertical axis indicates PI levels. (**C**) Quantitative analysis of DHME-induced CRC cell apoptosis. The annexin V-positive (apoptotic) cell population shown in (**B**) were scored. (**D**–**F**) Apoptosis blockade by z-VAD-fmk (50 μM) attenuates DHME-induced apoptosis as well as clonogenicity in CRC cells. The levels of the protein-to-GAPDH ratio relative to DHME-untreated controls were quantitated by ImageJ algorithm and are indicated below each blot. * *p* < 0.05; ** *p* < 0.01; *** *p* < 0.001.

To investigate whether the induction of apoptosis contributes to DHME-elicited CRC cytotoxicity, the levels of poly (ADP-ribose) polymerase (PARP) cleavage, a canonical hallmark of caspase activation and hence apoptosis, in DHME-treated CRC cells was evaluated by immunoblotting. We noted an evident, dose-dependent increase in cleaved PARP levels in all DHME-treated CRC cell lines, illustrating the induction of CRC cell apoptosis by DHME (Figure 3A). To further substantiate the proapoptotic effect of DHME on CRC cells, we examined the cell surface exposure levels of phosphatidylserine, which is another apoptosis hallmark and can be revealed by annexin V staining, in DHME-treated CRC cells using flow cytometry analysis. We found that DHME treatment led to a dose-dependent increase in the levels of the annexin V-positive (apoptotic) cell population (Figure 3B). Specifically, the apoptotic cell population of HCT 116 was 4.90% ± 1.05% at the basal level, and then dramatically increased to 47.17% ± 4.06% when treated with 20 μM of DHME (*p* < 0.001) (Figure 3C, top panel). Similarly, DHME at 20 μM provoked about five- and eight-fold increase in the apoptotic population of HCT-15 (from 14.23% ± 1.10% to 64.55% ± 3.28%, *p* < 0.001) and LoVo (from 9.3% ± 0.97% to 70.23% ± 3.15%, *p* < 0.001) cells, respectively (Figure 3C, middle and lower panels). To further assess the functional significance of apoptosis in the anti-CRC action of DHME, we tested the effect of z-VAD-fmk, a pan-caspase inhibitor, on DHME-induced cytotoxicity. It is noteworthy that in all CRC cell lines examined, the extents of both DHME-induced apoptosis and clonogenicity inhibition were markedly lowered when caspase activation was blocked by z-VAD-fmk (*p* < 0.001) (Figure 3D–F). Taken together, these results indicated that DHME-elicited CRC cytotoxicity was functionally attributed, at least in part, to the induction of apoptotic cell death.

### 2.4. DHME Suppressed WNT/β-Catenin Signaling in Human CRC Cells

**Figure 4:**
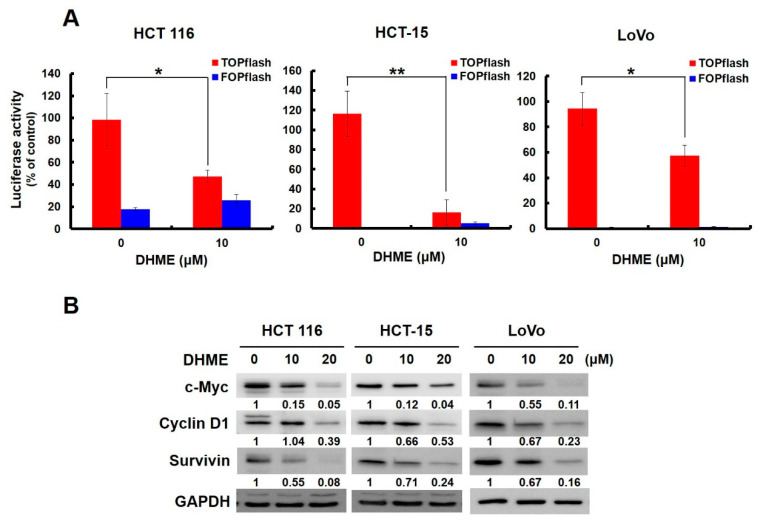
DHME inhibits WNT/β-catenin signaling in CRC cells. (**A**) Suppression of β-catenin–T-cell factor/lymphoid enhancer factor (TCF/LEF)-dependent transcription by DHME. HCT 116, HCT-15, and LoVo cells were transiently transfected with an M50 Super 8x TOPFlash plasmid (TOPFlash), a β-catenin luciferase reporter vector, followed by DHME treatment and then assessment of luciferase activity. The M51 Super 8x FOPFlash plasmid (FOPFlash) was used as a negative control for TOPFlash. * *p* < 0.05; ** *p* < 0.01. (**B**) DHME lowers the levels of c-MYC, cyclin D1, and survivin. Cell lysates of CRC cells, following 24 h treatment with DHME, were subjected to immunoblot analysis. GAPDH levels were used as the loading control. The levels of protein-to-GAPDH ratio relative to DHME-untreated controls were quantitated using ImageJ algorithm, and are indicated below each blot.

Given the fundamental contribution of aberrant WNT/β-catenin signaling to CRC pathogenesis and progression, we were eager to define the effect of DHME on this particular CRC oncogenic pathway using the TOPFlash assay system [19]. To this end, HCT 116, HCT-15, and LoVo cells transiently transfected with a luciferase-based β-catenin reporter plasmid (M50 Super 8x TOPFlash) were exposed to DHME (0 and 10 μM) for 24 h, and the status of the β-catenin–TCF/LEF-dependent transcription was then revealed by measuring the levels of luciferase activity. It is noteworthy that a marked decline in luciferase activity was observed in all of the tested CRC cell lines when treated with 10 μM of DHME (Figure 4A), suggesting that DHME suppressed WNT/β-catenin signaling in human CRC cells. Additionally, immunoblotting uncovered that DHME downregulated c-MYC, cyclin D1, and survivin, whose levels are known to be positively regulated by WNT/β-catenin signaling [20,21,22] (Figure 4B). Collectively, these results supported the inhibitory effect of DHME on WNT/β-catenin signaling in the context of CRC cells.

### 2.5. Suppression of WNT/β-Catenin Signaling was Essential for DHME to Induce CRC Cytotoxicity

**Figure 5:**
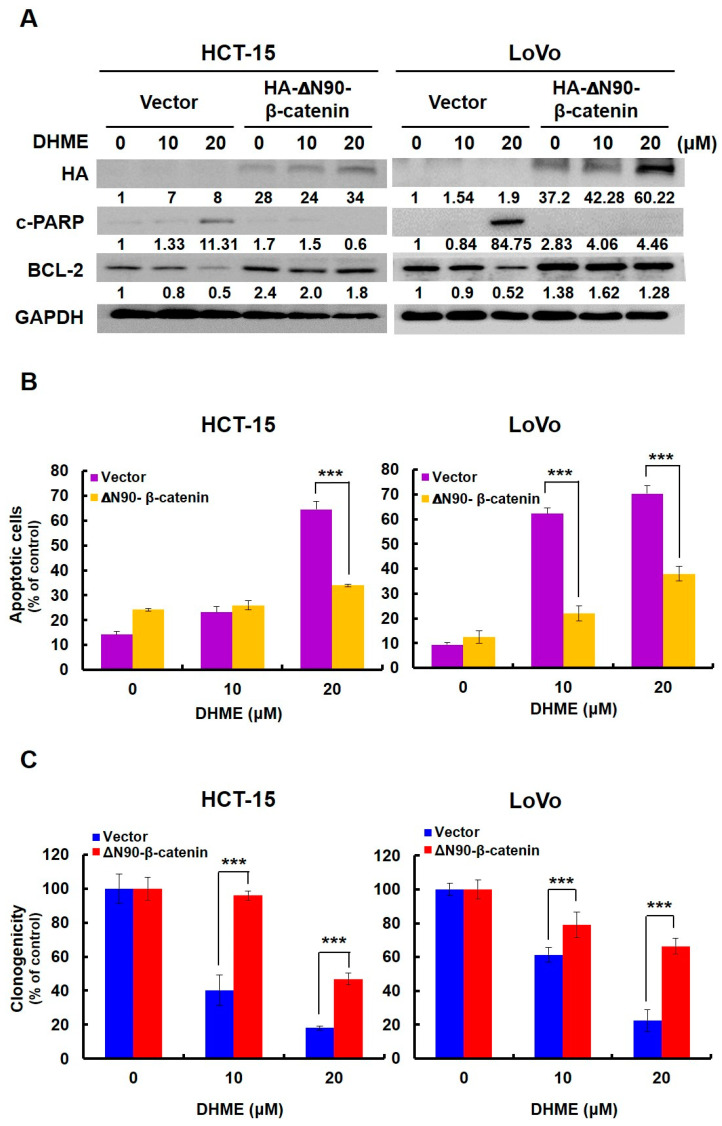
Blockade of WNT/β-catenin-mediated, pro-survival signaling is required for the anti-CRC action of DHME. (**A**) Persistent β-catenin activation abolishes DHME-induced PARP cleavage. HCT-15 and LoVo clones stably expressing HA–∆N90-β-catenin, an N-terminal, hemagglutinin (HA)-tagged, dominant-active β-catenin mutant (with N-terminal 90 amino acids deleted), were treated with DHME (0, 10, or 20 μM) for 24 h, followed by immunoblotting for the levels of HA (confirming ectopic expression of HA–∆N90-β-catenin), cleaved PARP (c-PARP), and BCL-2. GAPDH levels were used as the loading control. (**B**) Persistent β-catenin activation lowers the levels of the DHME-enhanced, annexin V-positive cell population. Stable vector or HA–∆N90-β-catenin clones of HCT-15 and LoVo cells were treated with DHME (0, 10, or 20 μM) for 24 h, followed by flow cytometry analysis for the levels of annexin V-positive (apoptotic) cell population. (**C**) Persistent β-catenin activation rescued DHME-mediated inhibition of clonogenicity. A total of 2 × 10^2^ of stable vector or HA–∆N90-β-catenin clones of HCT-15 and LoVo cells after 24 h treatment of DHME (0, 10, or 20 μM) were assessed for their ability to form colonies. The levels of protein-to-GAPDH ratio relative to DHME-untreated vector controls were quantitated by ImageJ algorithm and are indicated below each blot. *** *p* < 0.001.

We next addressed the functional significance of WNT/β-catenin signaling blockage in DHME- elicited anti-CRC action. In this regard, CRC cell lines stably expressing a constitutively active β-catenin mutant (β-catenin with its N-terminal 90 amino acids deleted (∆N90-β-catenin) [23,24]) were generated to withstand DHME-induced blockade of WNT/β-catenin signaling. It is noticed that DHME failed to provoke evident PARP cleavage in ∆N90-β-catenin stable clones, in contrast to DHME-treated vector controls, whose cleaved PARP levels were obviously elevated (Figure 5A). Also, it is noteworthy that DHME-induced PARP cleavage was inversely correlated with the levels of antiapoptotic BCL-2. In detail, DHME lowered BCL-2 levels in vector control clones, whereas constitutive β-catenin activation prevented BCL-2 from DHME-induced downregulation, likely contributing to the attenuation of PARP cleavage (Figure 5A). Thus, these data together argued that blockade of the WNT/β-catenin-BCL-2 pro-survival signaling axis is essential for the proapoptotic action of DHME. In parallel to the immunoblotting evidence, we further unveiled a marked drop in the levels of the annexin V-positive cell population (Figure 5B), in addition to enhanced clonogenicity (Figure 5C) in ∆N90-β-catenin stable clones, compared to their respective vector controls (*p* < 0.001). Altogether, our findings identified suppression of WNT/β-catenin signaling as an integral mechanism of action of DHME-induced CRC cytotoxicity.

## 3. Discussion

In this study, the DHME-induced, pro-apoptotic effect and the mechanism of DHME’s pro-apoptotic action in the context of human CRC cell lines was delineated for the first time. Specifically, we began by showing the cytotoxic effect of DHME on CRC cells while sparing normal colon epithelial cells (Figure 1), and further revealed that DHME was more potent than hispolon with respect to triggering CRC cell death (Figure 2). In addition, we verified that DHME-evoked CRC cytotoxicity was largely attributed to the induction of apoptosis-dependent cell death (Figure 3). Moreover, we identified that the aberrantly active WNT/β-catenin signaling in CRC cells was suppressed upon DHME stimulation (Figure 4), and notably, the ectopic expression of a dominant–active β-catenin mutant (∆N90-β-catenin) abolished DHME-induced CRC cytotoxicity, likely due to sustained BCL-2 expression in the context of constitutive β-catenin activation (Figure 5). To our best knowledge, the discoveries regarding the selective cytotoxicity of DHME against malignant colorectal epithelial cells, in addition to the blockade of WNT/β-catenin signaling as the mechanism of DHME-induced cytotoxic action on CRC cells, have never been documented.

The data presented here indicate that DHME was cytotoxic against a panel of human CRC cell lines and exhibited stronger CRC cytotoxicity than hispolon (Figure 1 and Figure 2). It is noted that Balaji et al. have studied the in vitro cytotoxicity of hispolon and various hispolon derivatives, including DHME (designated as compound V5), in two representative cell lines from human breast cancer, CRC, and prostate cancer [18]. Intriguingly, in that report hispolon was shown to be more cytotoxic than DHME against HCT 116 cells. The apparent discrepancy between the results of Balaji et al. and ours is puzzling. One possible explanation is the different experimental settings of cell-based cytotoxicity assays used in the studies, where different numbers of cells subjected to drug treatment (5 × 10^3^ in Balaji et al. vs. 8 × 10^3^ in ours), as well as different length of drug incubation time (72 h in Balaji et al. vs. 48 h in ours) were applied. Alternatively, there might be certain disparities in the HCT 116 cell line between the one used by Balaji et al. and ours, such as growth status and passage numbers, which likely led to different sensitivity of cells to drug treatment.

In this study, we demonstrated that DHME is pro-apoptotic to CRC cells, and further confirmed the induction of CRC cell apoptosis as a central mechanism underlying DHME-mediated cytotoxic action (Figure 3). Still, we noticed that blockade of apoptosis by pan-caspase inhibitor z-VAD-fmk did not completely abolish DHME-induced CRC cytotoxicity, suggesting the involvement of additional mechanisms of DHME-mediated cytotoxic action. It is noteworthy that hispolon has been revealed to provoke autophagy in addition to eliciting apoptosis [16,25]. For that reason, it would be necessary to explore the contribution of autophagic death to DHME-induced cytotoxic action against CRC cells.

Mounting evidence has underscored the fundamental role of aberrant WNT/β-catenin signaling in CRC genesis and malignant progression. It is well-established that WNT/β-catenin signaling controls pleiotropic events of malignancy, including sustaining stemness and promoting cell proliferation, survival, and invasion [5]. Along this line, our discovery that DHME functions as a potent inhibitor of aberrant WNT/β-catenin signaling in CRC cells (Figure 4) paves the way for clinical translation of DHME into CRC therapeutics. Still, the mechanisms whereby DHME blocks WNT/β-catenin signaling in CRC cells currently remain elusive. It is noteworthy that in most CRC cells, the aberrantly active WNT/β-catenin signaling is not attributed to upstream WNT signals, as observed in other human cancers, but rather caused by the constitutive activation of β-catenin owing to loss-of-function mutations in the *APC* gene, as seen in HCT-15 and LoVo cells [26,27] or gain-of-function mutations in the β-catenin-encoding *CTNNB1* gene, as found in HCT 116 cells [28]. The resultant, constitutively active β-catenin in turn upregulates TCF/LEF-dependent transcription of WNT target genes [5,6]. Therefore, DHME-mediated inhibition of WNT/β-catenin signaling in these CRC cell lines is most likely achieved at the level of β-catenin–TCF/LEF-dependent transcription. Thus, addressing how DHME sabotages β-catenin–TCF/LEF-dependent transcription is necessary to understand the pharmacology of DHME-mediated anti-CRC activity, and is currently under investigation in our laboratory.

As a central regulator of cell survival, WNT/β-catenin signaling is known to inhibit apoptosis by activating β-catenin/TCF-mediated transcription of antiapoptotic genes, such as BCL-xL and survivin [29,30], or by inactivating proapoptotic BAX via a PI3K/AKT-dependent mechanism [31]. In this study, we unraveled BCL-2 as a possible downstream effector of WNT/β-catenin signaling to promote CRC cell survival, as evidenced by the resistance of BCL-2 to DHME-mediated downregulation, along with DHME-induced apoptosis when β-catenin was constitutively active (Figure 5). It is worth noting that while in this study, no direct evidence was presented to denote BCL-2 as a target gene of WNT/β-catenin signaling in CRC cells, previous studies have proven a direct binding of TCF4 to the promoter of the *BCL-2* gene in human CRC cell lines HT-29 and SW620 [32,33]. In view of that, it is plausible to argue that DHME triggers CRC cell apoptosis by downregulating BCL-2 through the inhibition of WNT/β-catenin signaling.

**Figure 6:**
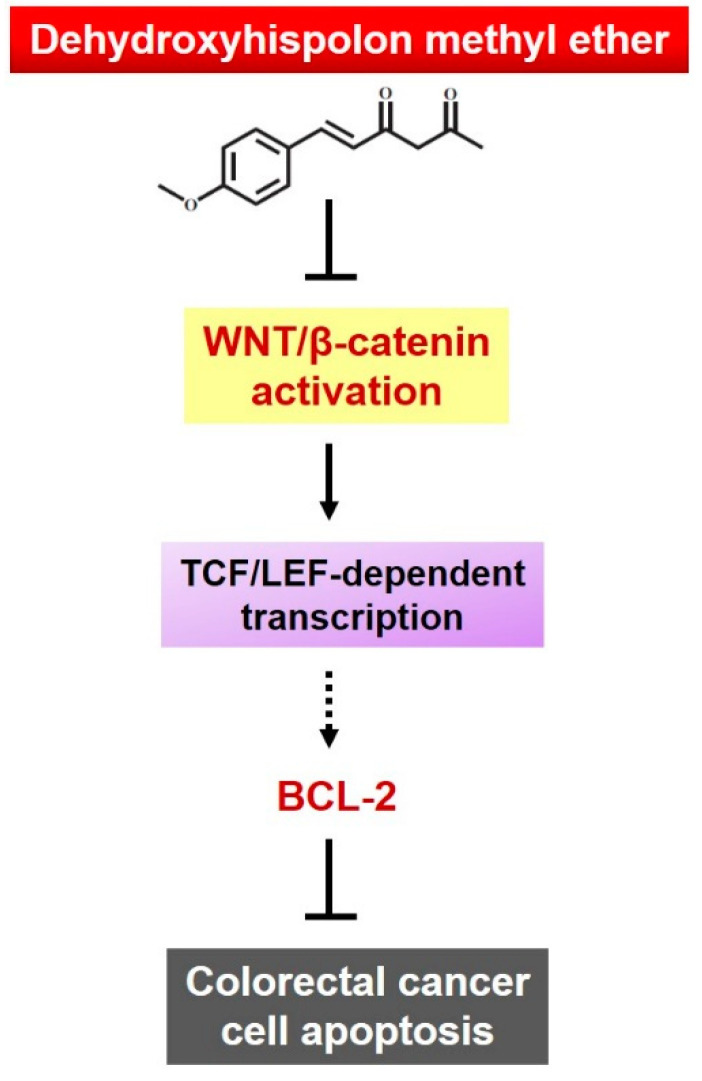
Schematic diagram depicting the anti-CRC mechanism of action of DHME elucidated in this study. In brief, DHME induces CRC cell apoptosis via the targeted inhibition of the pro-survival WNT/β-catenin–BCL-2 signaling axis. The chemical structure of DHME is adapted from Balaji et al. [18]. The dashed line denotes that our data implicated that the transcription of *BCL-2* likely depends on the β-catenin–TCF/LEF transcription complex, but it still requires evidence to support the direct binding of TCF/LEF to the human *BCL-2* promoter for driving *BCL-2* transcription in the CRC cell lines used in this study.

In conclusion, we herein present the first report that documents DHME’s selective cytotoxicity to CRC cells while sparing normal colon epithelial cells, reveals a stronger CRC cytotoxicity of DHME than that of its parental structure hispolon, and establishes the mechanism of DHME-elicited cytotoxic action involving the induction of CRC cell apoptosis via suppression of the WNT/β-catenin signaling axis (Figure 6). Collectively, our discovery highlights the potential to translate DHME into a CRC therapeutic regimen, but also warrants the investigation of additional hispolon derivatives regarding their anticancer perspectives.

## 4. Materials and Methods

### 4.1. Chemicals

Dehydroxyhispolon methyl ether (DHME) and hispolon were chemically synthesized and prepared as reported in Balaji et al. [18]. Pan-caspase inhibitor z-VAD-fmk was purchased from Cayman Chemical (Ann Arbor, MI, USA), prepared as a 50 mM stock solution in dimethyl sulphoxide (DMSO) (Sigma-Aldrich; St. Louis, MO, USA), and stored at 4 °C until use.

### 4.2. Plasmids

The luciferase reporter vector for β-catenin-mediated, TCF/LEF-mediated, transcriptional activity M50 Super 8x TOPFlash (Addgene plasmid #12456) and the TOPFlash mutant vector M51 Super 8x FOPFlash (Addgene plasmid #12457) were gifts from Professor Randall Moon. The pBabe-HA–∆N90-β-catenin plasmid, a pBabe-based vector for the ectopic expression of an N-terminal, hemagglutinin (HA) epitope-tagged, dominant–active β-catenin mutant, has been described in detail in our previous report [34]. Production of pBabe-derived retroviral particles and subsequent infection to target cells was performed according our established protocols [35].

### 4.3. Cell culture

Human colorectal carcinoma (CRC) cell lines HCT 116 (ATCC CCL-247), HCT-15 (ATCC CCL-225), and LoVo (ATCC CCL-229) were obtained from the Bioresource Collection and Research Center (BCRC) (Hsinchu, TWN), and were cultured in McCoy’s 5a, RPMI-1640, and F-12K media (Gibco Life Technologies; Carlsbad, CA, United States), respectively. Normal human colon epithelial cell line CCD 841 CoN (ATCC CCL-1790) was purchased from the American Type Culture Collection (ATCC) (Manassas, VA, USA), and was grown in Eagle’s Minimum Essential Medium (MEM) medium. All of the culture media were replenished with 10% fetal bovine serum, 1% penicillin–streptomycin, and 1% sodium pyruvate (Gibco Life Technologies; Carlsbad, CA, USA). Cells were grown at 37 °C in a humidified environment with 5% CO_2_.

### 4.4. In vitro Cytotoxicity Analysis

The in vitro cytotoxicity of DHME and hispolon was determined by the levels of cell viability after drug treatment for 48 h by the use of a CellTiter 96 AQueous One Solution Cell Proliferation Assay (MTS assay) (Promega; Madison, WI, USA), in addition to evaluating the capacity of drug-treated cells to form colonies (clonogenicity assay). Assays for cell viability and clonogenicity were conducted following our established protocol [35,36].

### 4.5. Immunoblotting

Immunoblotting was carried out as previously reported [36]. Primary antibodies against BCL-2 (#2872), c-MYC (#13987), HA epitope (#3724), and cleaved PARP (#9541) were purchased from Cell Signaling Technology (Boston, MA, United States). Anti-cyclin D1 (GTX108624), anti-GAPDH (GTX627408), and anti-survivin (GTX100052) polyclonal antibodies were obtained from GeneTex (Hsinchu, TWN). Secondary antibodies against rabbit IgG (#111-035-003) were bought from Jackson ImmunoResearch (West Grove, PA, USA).

### 4.6. Apoptosis Analysis

DHME-induced apoptosis was quantitatively evaluated by flow cytometry analysis to identify the levels of annexin V-stained (apoptotic) cells on the Muse Cell Analyzer, using Muse annexin V and a Dead Cell Assay Kit (Millipore; Burlington, MA, USA), in accordance with the procedures reported previously [35]. To elucidate the significance of apoptosis induction to DHME-induced cytotoxicity, CRC cells were pre-treated for 1 h with 50 μM of z-VAD-fmk to block apoptosis, followed by 24 h incubation with DHME for subsequent apoptosis analysis.

### 4.7. WNT/β-catenein Signaling Analysis

The effect of DHME on the activity of WNT/β-catenein signaling was determined by the luciferase-based TOPFlash reporter system, where the amount of β-catenein-mediated TCF/LEF-dependent transcription activity was revealed by the activity of the luciferase reporter. A plasmid expressing *Renilla* luciferase (Promega; Madison, WI, USA) was transfected into cells along with TOPFlash or FOPFlash plasmids for normalization of transfection efficiency. Dual luciferase activity assay was executed in accordance to our established protocol [36].

### 4.8. Statistical analysis

All data were obtained from at least three independent experiments, and are expressed as means ± SD. Unpaired, two-tailed *t*-tests were employed to determine the difference between two independent experiments, and the difference was considered statistically significant when *p* < 0.05.

## Abbreviations

APC	Adenomatous polyposis coli
BCL-2	B-cell lymphoma 2
EGFR	Epidermal growth factor receptor
MAPK	Mitogen-activated protein kinase
PI3K/AKT	Phosphoinositide 3-kinases/ Protein Kinase B
PARP	Poly (ADP-ribose) polymerase
TGF-β	Transforming growth factor-β
TCF/LEF	T-cell factor/lymphoid enhancer factor
